# An Innovative Approach for Elemental Mercury Adsorption Using X-ray Irradiation and Electrospun Nylon/Chitosan Nanofibers

**DOI:** 10.3390/polym16121721

**Published:** 2024-06-17

**Authors:** Baturalp Yalcinkaya, Martin Strejc, Fatma Yalcinkaya, Tomas Spirek, Petr Louda, Katarzyna Ewa Buczkowska, Milan Bousa

**Affiliations:** 1 Department of Material Science, Faculty of Mechanical Engineering, Technical University of Liberec, Studentska 2, 461 17 Liberec, Czech Republic; petr.louda@tul.cz (P.L.); katarzyna.ewa.buczkowska@tul.cz (K.E.B.); milan.bousa@tul.cz (M.B.); 2 ÚJV Řež, a. s., 250 68 Husinec, Czech Republic; martin.strejc@cvrez.cz; 3 Faculty of Mechatronics, Institute for New Technologies and Applied Informatics, Technical University of Liberec, Studentska 1402/2, 461 17 Liberec, Czech Republic; fatma.yalcinkaya@tul.cz; 4 Green Energy Consulting, s.r.o., Pod Altánem 9/105, 100 00 Prague, Czech Republic; tomas.spirek@gec-gec.cz; 5 Faculty of Mechanical Engineering, University of Kalisz, ul. Nowy Świat 4, 62-800 Kalisz, Poland

**Keywords:** polyamide, chitosan, nanofibers, electrospinning, mercury filtration, X-ray irradiation, innovative development, sustainable production

## Abstract

A novel approach was proposed, utilizing an electrical field and X-ray irradiation to oxidize elemental mercury (Hg^0^) and encapsulate it within a nanofibrous mat made of Polyamide 6/Chitosan. The X-rays contributed significantly to the conversion of Hg^0^ into Hg^+^ by producing electrons through the photoionization of gas molecules. The positive and negative pole electrodes generated an electric field that exerted a magnetic force, resulting in the redirection of oxidized elemental mercury towards the negative pole electrode, which was coupled with a Polyamide 6/Chitosan nanofiber mat. The evaluation of the Polyamide 6/Chitosan nanofibers exposed to oxidized mercury showed that the mercury, found in the steam of a specially designed filtration device, was captured in two different forms. Firstly, it was chemically bonded with concentrations ranging from 0.2 to 10 ng of Hg in total. Secondly, it was retained on the surface of the Polyamide 6/Chitosan nanofibers with a concentration of 10 microg/m^3^ of Hg per minute. Nevertheless, a concentration of 10 microg/m^3^ of mercury is considered significant, given that the emission levels of mercury from each coal power plant typically vary from approximately 4.72 to 44.07 microg/m^3^. Thus, this research presents a viable approach to reducing mercury emissions from coal-fired power plants, which could result in lower operational expenses and less secondary environmental effects.

## 1. Introduction

Coal power plants and coal combustion industries are responsible for various environmental problems that can significantly impact human health and the natural world [1,2,3]. One of the most pressing issues is air pollution. Burning coal releases multiple pollutants, such as sulfur dioxide, nitrogen oxides, particulate matter, and mercury [4]. These pollutants can cause respiratory problems and other health issues, contributing to various negative impacts on human health [5,6]. Additionally, coal combustion emits large amounts of carbon dioxide. This greenhouse gas contributes to climate change, which poses a significant threat to ecosystems and societies worldwide [7]. Coal-fired power generation can contaminate water sources with toxic chemicals, such as arsenic, lead, and mercury [8,9,10].

Mercury emitted from coal-fired power plants can be classified into three types: elemental mercury (Hg^0^), gaseous oxidized mercury (Hg^+^), and particle-bound mercury (HgP) [11,12]. All of these will have an immediate or potential negative impact on human health. Currently, most coal-fired power plants release 5 kg of mercury annually. However, there are specific groups of power plants that burn lignite in North Dakota and Texas, which emit over 100 kg of mercury each year [13]. As a result, understanding how to remove mercury from coal-fired power plants is critical, as is a study into mercury speciation and emissions. There are several existing technologies for mercury filtration in coal-fired power plants. Some of the most commonly used technologies include activated carbon injection (ACI) [14,15], wet scrubbers, fabric-based filters, dry sorbent injection (DSI) [16,17], flue gas desulfurization (FGD) [18,19], and selective catalytic reduction (SCR) [20]. Each of these technologies has its advantages and disadvantages, and the choice of technology depends on factors such as the coal being burned, the power plant’s configuration, and the local regulatory requirements. In addition, installation, maintenance, cleaning, storage, or disposal of waste and activated carbons all contribute to an increase in the cost of the mercury filtration systems that were previously stated [21,22].

Chitosan is a biopolymer derived from chitin, which is commonly found in the exoskeletons of crustaceans like shrimp and crabs. The nanofiber form of chitosan [23,24] has a high surface area-to-volume ratio, making it an ideal adsorption material [25,26,27,28]. When chitosan nanofibers are exposed to mercury-contaminated water, the mercury ions bind to the chitosan through a process called adsorption. Once the mercury ions are bound, they are effectively removed from the water, thus purifying it [29].

Using chitosan nanofibers for mercury removal has been proved in wastewater treatment. In a study conducted by researchers [30], chitosan nanofibers were incorporated into a filter to remove mercury from simulated wastewater. The results showed that the chitosan nanofiber filter could remove up to 99% of the mercury in the wastewater. Another example is in the treatment of contaminated soil. Chitosan nanofiber mats were used to remove mercury from contaminated soil [31]. The chitosan nanofiber mats were placed on top of the contaminated soil and left for a period of time. The results showed that the chitosan nanofibers were able to significantly reduce the concentration of mercury in the soil. Overall, chitosan nanofibers have demonstrated outstanding potential as a cost-effective and environmentally friendly material for removing mercury from contaminated water and soil.

It is not feasible to create nanofibers using the electrospinning technique with a polymer solution consisting solely of a chitosan polymer. Hence, combining chitosan with other natural or synthetic polymers is essential to enhance its ability to be spun into fibers [25]. The selection of the optimal co-polymer for chitosan largely hinges on the intended application of the resulting nanofibers. Chitosan can be combined with gelatin [32], polylactic acid [33], and polycaprolactone [34] for tissue engineering purposes. It can also be blended with polyvinyl alcohol [35,36], polyethylene oxide [37], cellulose acetate [38], and polyacrylonitrile [39] for use in active food packaging and antibacterial activity. Polyvinylpyrrolidone [40] and polyimide [41] are utilized to adsorb pollutants.

As per our knowledge, this study represents the first analysis of its kind by utilizing X-ray irradiation to convert atomic mercury (Hg) to its ionized form (Hg^+^), which was then adsorbed by Polyamide 6/Chitosan (PA6/CS) nanofibers through the incorporation of an electrical field and negatively charged electrode. The system operated in harmony, as evidenced by the mercury detection sensor. The adsorbed mercury on the PA6/CS nanofibers was analyzed through the use of a mercury analyzer AMA 254. The suggested prototype successfully proved that complex systems, such as the chitosan nanofibrous combination with X-ray irradiation and electrical forces, can effectively reduce emitted toxic mercury pollution from coal power plants.

## 2. Materials and Methods

### 2.1. Experimental Materials

Ultra-aramid B24 polyamide 6 was used as a co-polymer and procured from BASF, Limburgerhof, Germany. At the same time, the medium-molecular-weight chitosan was obtained from Sigma-Aldrich, Saint Louis, MO, USA. The PA6/CS polymer solution was created in accordance with a prior experiment [25]. Liquid mercury was purchased from Merck (Darmstadt, Germany) and utilized in the feeding gas to carry out the adsorption tests in the gas chamber. The solvents, namely 99% acetic acid (AA) and formic acid (FA), were obtained from Penta s.r.o. Prague, Czech Republic.

### 2.2. Preparation of Polyamide 6/Chitosan Nanofibers

**Figure 1:**
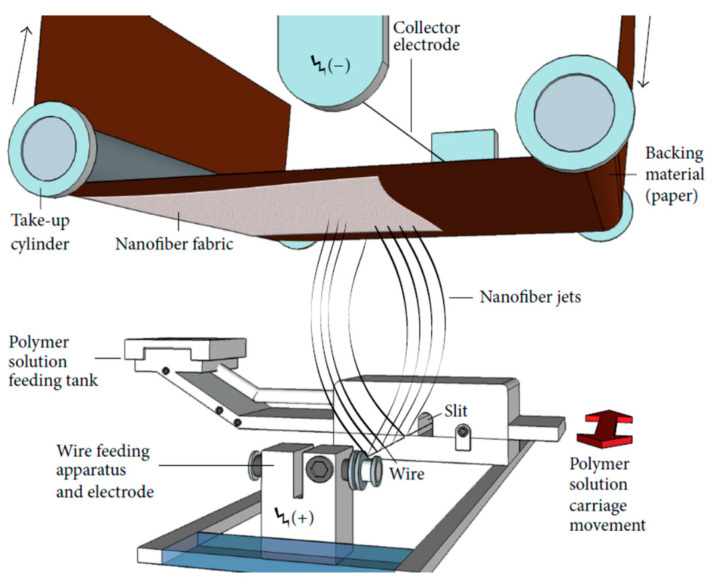
Laboratory size of Nanospider electrospinning equipment [42].

Initially, polyamide 6 granules were dissolved in acetic acid and formic acid, and the resulting solution was mixed for 4 h at a temperature of 60 °C. Subsequently, chitosan powder was added to the solution and mixed for an additional 4 h. The nanofibers were fabricated through the electrospinning method, using a commercial electrospinning device called the Nanospider NSLAB, developed by Elmarco s.r.o, Liberec, Czech Republic (Figure 1). In nanospider electrospinning, the PA6/CS polymer solution is extruded on a wire spinneret containing hundreds of fine jets, creating a web-like structure. The spinneret is placed in an electric field, and a high voltage is applied to the polymer solution. The electric field causes the solution to stretch and elongate, forming thin PA6/CS nanofibers that is then collected on a backing paper substrate at a rate of 2 g per square meter. One of the advantages of nanospider electrospinning is the ability to produce high-quality nanofibers with a high level of control over fiber diameter morphology. The solution and process parameters are outlined in Table 1.

### 2.3. Characterization and Evaluations of Nanofibers

Field emission scanning electron microscopy (FE-SEM), Carl Zeiss ULTRA Plus, Jena, Germany, was employed to observe the surface morphology of PA6/CS nanofibers. The microscope has the following parameters: resolution: 1 nm @ 15 kV; 1.6 nm @ 1 kV; magnification: 12–1,000,000× in SE mode; and acceleration voltage: 0.02–30 kV. The fiber diameter was measured using ImageJ 1.54g software. Elemental analysis of the prepared nanofibers was performed using electron microscopy and the EDS method. Energy-dispersive spectrometry (EDS, elemental analysis Jena, Germany) is an analytical method based on detecting the characteristic X-ray radiation created by the interaction of an electron beam in a microscope with the surface of the examined sample. Fourier transform infrared spectroscopy (Nicolet iZ10 a Nicolet iN10 MX, Thermo Scientific, Brno, Czech Republic) was used to analyze the chemical composition and crystallinity of all samples with a range of 400–4000 cm^−1^.

### 2.4. Mercury Adsorption Test

**Figure 2:**
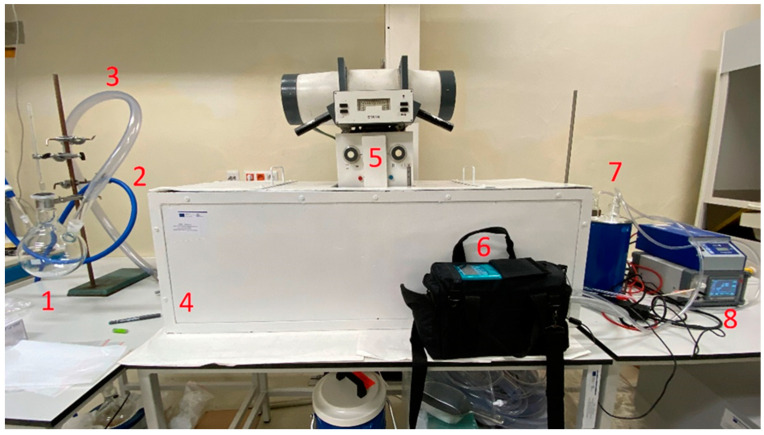
Custom-made mercury adsorption device.

In order to capture mercury, a custom-made device was built (Figure 2). Specialists at the designated facility ÚJV Řež, a. s., Prague, Czech Republic, conducted all tests and investigations relevant to mercury. The system comprised several components. The experimental setup included an industrial-grade pressurized nitrogen source, a three-neck round bottom flask, an X-ray source, a lead chamber, a Hg detector, a cooling flask with CO_2_ dry ice, and an air outlet ventilation. In total, 15 g liquid mercury was placed into the three-neck round bottom flask (1). The nitrogen gas source was connected to one of the openings of the flask (2), and the other opening (3) was connected to the inlet of the main body of the device (4). The main body (4) was made entirely from lead to prevent the outward radiation of X-rays. The source of X-rays (Chirana Praha, Czech Republic, type: MP15) (5) was positioned on the top of the main body and directed to the main flow of nitrogen/mercury gases. The gas steam vented out from the main body of the device, and steam was analyzed by a Hg detector (6) (Lumex Instruments LIGHT-915, Fraserview, Vancouver, BC, Canada); data were sent to the computer simultaneously. Another flask, immersed in dry ice, was positioned at the end of the main flow of nitrogen/mercury to retain non-adsorbed mercury (7). The power source (8) to the electrodes was provided by the power generator (Simco-ION CM Lite, Kilkenny, Ireland).

**Figure 3:**
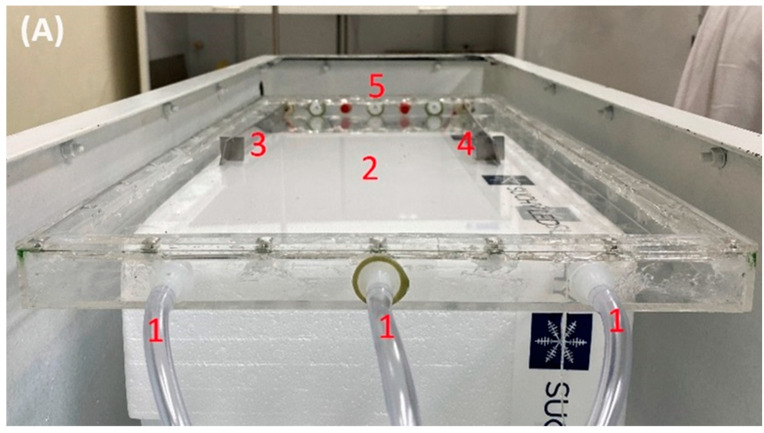

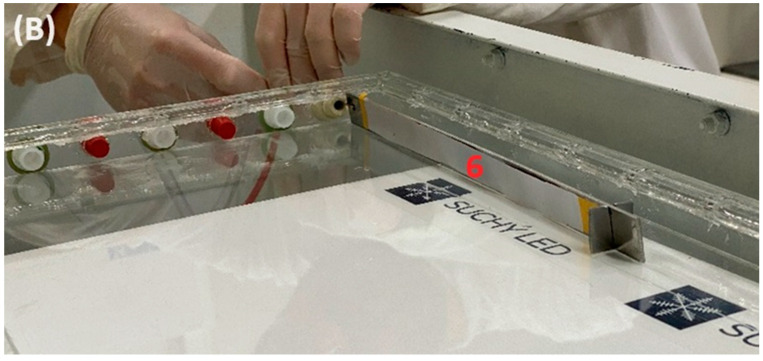
Adsorption cell inside the lead main body (**A**) and negative poll electrode with nanofibrous layer (**B**).

The adsorption cell was placed into the lead main body (Figure 3A). Nitrogen gas with an evaporated mercury inlet was connected to the cell (1), and the X-ray source was positioned to be exposed to the area numbered (2). At the same time, electrodes numbered (3) (positive poll) and (4) (negative poll) were positioned horizontally to create an electrical field to attract Hg^+^. A 10 cm^2^ PA6/CS nanofiber mat was placed on the surface of electrode (4) and is marked as (6) in Figure 3B. Unretained mercury vapor was released from the cell through outlet pipes (5).

During the initial stage of the tests, it is crucial to maintain a stable mercury concentration in the system for 25 min. Following stabilization, the X-ray source was activated, and a voltage was delivered between the electrodes for 20 min. Subsequently, the X-ray source was turned off and cooled. The chamber was thoroughly flushed with nitrogen for safety purposes. The experiment was utilized in certain conditions, including an unheated mercury flask at room temperature (22 °C), a gas flow rate of 5 L per minute, and an electrode voltage of 10 kV. The quantity of mercury utilized in the experiment ranged from approximately 270 micrograms per cubic meter. The emission concentration of mercury from coal power plants worldwide ranges from roughly 4.72 to 44.07 micrograms per cubic meter [43].

### 2.5. Measurements of Mercury Content Adsorbed to the PA6/CS Nanofibers

The AMA-254, developed by Altec, Ltd., Prague, Czech Republic, is a specialized atomic absorption spectrometer used to detect mercury on nanofibers. The operation of the instrument can be divided into three phases for each analysis: decomposition, collection, and detection. The samples of PA6/CS nanofibers exposed to mercury gases are subjected to thermal treatment upon introduction into the analyzer. The substance is first dehydrated and subsequently ignited in the presence of an oxygen flow. The combustion gases undergo decomposition in the catalytic column. The mercury vapor is efficiently captured on the surface of a gold amalgamator. Subsequently, the amalgamator is subjected to heating at approximately 700 °C in order to release mercury towards the detection system, which incorporates a Hg-specific lamp that emits light at a wavelength of 253.7 nm. A silicon ultraviolet diode detector is utilized for the measurement of mercury contents. The operational range (with automatic transition for lower and higher concentrations of Hg) is 0.05–500 ng Hg.

## 3. Results and Discussion

### 3.1. Characterization of PA6/CS Nanofibers

**Figure 4:**
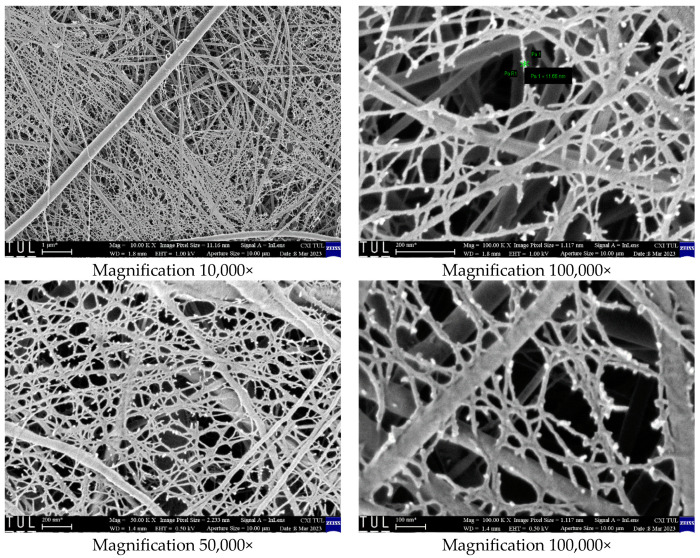
SEM images of PA6/CS nanofibers. “*” means an approximate value.

Figure 4 illustrates the SEM images of electrospun nanofibers. Polyamide and chitosan polymers were initially homogeneously mixed to form a polymer solution. Following the electrospinning of a well-dispersed polymer solution, the fibers that were produced displayed an interlinked structure that was one of a kind. A support network was formed by polyamide fibers with a diameter ranging from 80 to 100 nanometers, and this network was consistent with the network formed by pure PA6 fibers that were generated under the same conditions. On the other hand, chitosan fibers came together to form a fine web that was intricately related to the PA6 fibers system. More specifically, the chitosan fibers had a diameter of approximately 10 nanometers, which was a considerable reduction in thickness. In the illustration on the right-hand side, the diameter of a specific chitosan fiber is depicted in green for the sake of clarity. This particular fiber has a diameter of 11.7 nanometers.

The low concentration of chitosan in the polymer solution structure is the cause of the formation of chitosan nanofibers with a low fiber diameter. The matrix exhibits strong spinnability and covering performance. The polyamide 6 polymer’s high compatibility with the electrospinning method facilitated the formation of high-quality nano-sized chitosan fibers. The importance of choosing polyamide 6 nanofibers over other polymers is due to its extremely versatile spinnability, high throughput, good mechanical properties, and relatively high-temperature tolerance. The filters in a flue gas environment are subjected to high temperatures in real-time settings [44,45]. The TGA findings in the literature indicate that the polyamide 6 nanofiber, both on its own and when blended with chitosan polymers, exhibits exceptional resistance up to a temperature of 400 °C [46,47].

**Figure 5:**
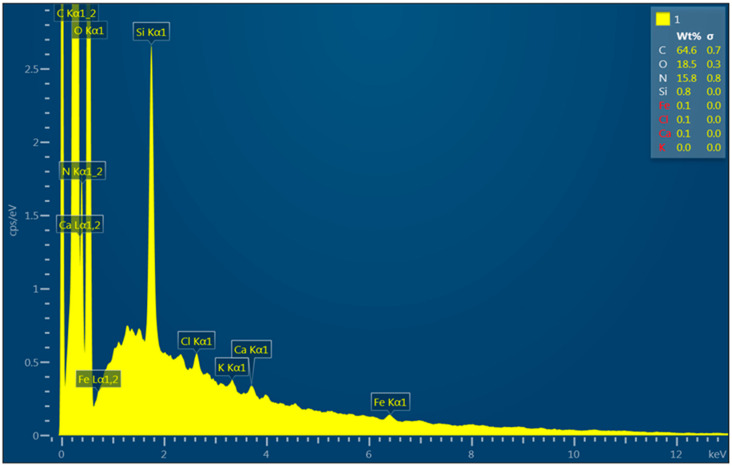
EDS analysis of PA6/CS nanofibers.

Figure 5 and Table 2 show the elemental analysis of the prepared PA6/CS nanofibers. In addition to the content of carbon and oxygen (organic structure of polymers), nitrogen, which is in the structure of chitosan, is also detected. Silicon, which is contained in the backing paper, is also represented. The other elements marked in red color can be neglected due to their minimal representation of around 0.1 wt.% and an uncertain calculation close to the detection limit.

**Figure 6:**
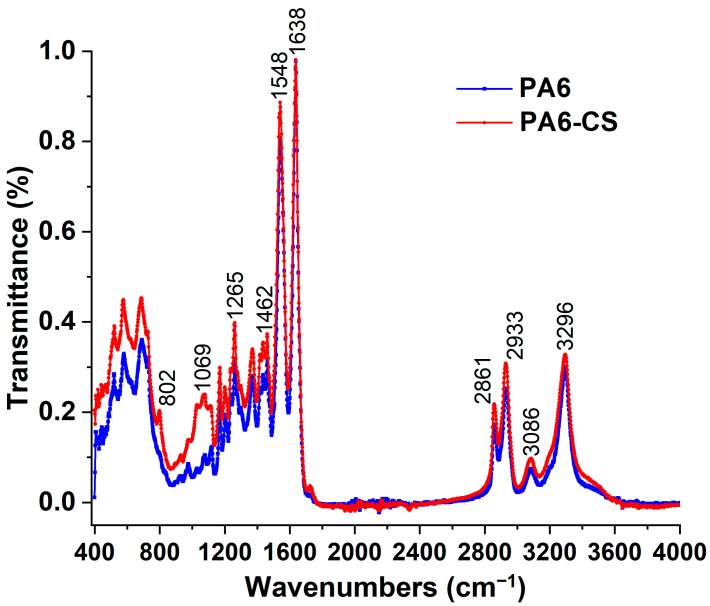
FT-IR analysis and comparison of PA6 and PA6/CS nanofibers.

The interactions between polyamide and chitosan were investigated using the FT-IR spectroscopy technique in a range from 400 to 4000 cm^−1^, as shown in Figure 6. FT-IR is a well-defined method for detecting intermolecular interactions between two polymers, encompassing the identification of new functional groups formed through chemical reactions and the characterization of intermolecular interactions facilitated by hydrogen bonding. The FT-IR spectra of PA6/CS were compared to pristine PA6 nanofibers.

The transmittance peaks indicative of the characteristic properties of polyamide 6 nanofibers can be attributed as follows [48,49,50]: 3296 cm^−1^ (stretching of -NH groups), 3086 cm^−1^ (N–H in-plane bending), 2933 cm^−1^ (antisymmetric stretching vibration of CH_2_), 2861 cm^−1^ (symmetric stretching vibration of CH_2_), 1638 cm^−1^ (amide I γ CO), 1548 cm^−1^ (amide II δN–H), 1462 cm^−1^ (in-plane deformation of CH_2_), 1265 cm^−1^ (CH_2_ twist-wag vibration), and 688 cm^−1^ (out-of-plane deformation N–H and C=O).

Compared with the FT-IR spectra for pristine PA6 nanofibers, the new absorption peak of the PA6/CS blended nanofibers was observed at 1069 and 802 cm^−1^, which might indicate the saccharine geometry of chitosan molecules [51] and wagging of the saccharide structure of chitosan [52]. The FT-IR results confirm the presence of CS in the PA6 nanofibers.

### 3.2. Adsorption of Elemental Mercury

**Figure 7:**
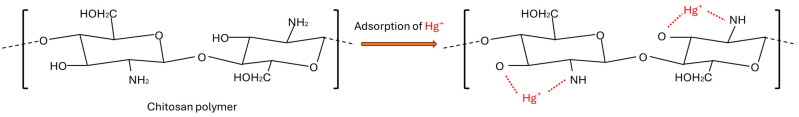
Chemical structure of Hg^+^ adsorption by chitosan nanofibers.

The adsorption of Hg^+^ ions by chitosan is believed to happen through several individual or combined interactions, primarily involving the amino group (-NH_2_) and hydroxyl (-OH), as shown in Figure 7. These interactions result in the adsorption of Hg^+^ ions via chelation, ion exchange, or electrostatic forces [53,54].

**Figure 8:**
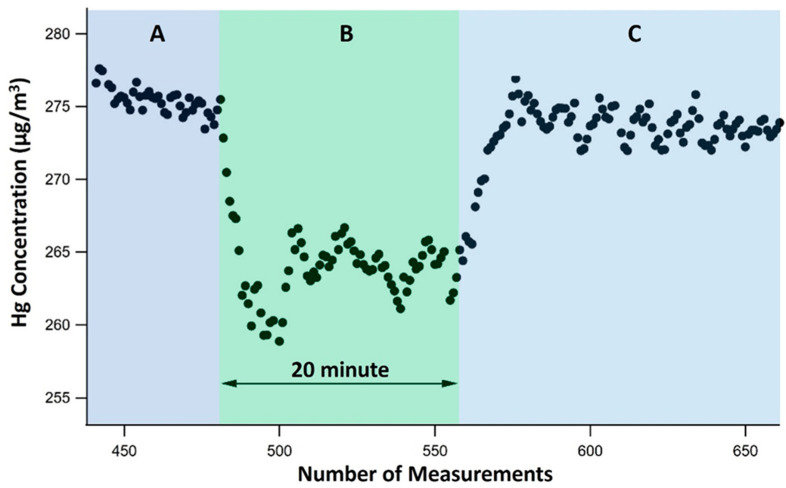
Concentration of mercury in the filtering systems. Section A: stabilization period. Section B: X-ray and power supply ON elemental Hg filtration. Section C: X-ray and power supply OFF.

The graph in Figure 8 shows the course of the adsorption experiment. Section A corresponds to the initial stabilization period of the experiment, during which the presence of mercury in the flow was first observed at levels ranging from 275 to 278 micrograms per cubic meter.

Section B is designated for the 20 min activation of the X-ray and electric field (power generator). The mercury analyzer at the chamber outlet detected a noticeable reduction in the mercury concentration, averaging 4.4%.

The sudden drop in the mercury concentration can be explained based on the ionization of mercury atoms by X-rays and their subsequent separation by an electromagnetic field and the trapping by the PA6/CS nanofiber placed on the negatively charged electrode in the form of a metal plate. The used X-rays are a spectrum of photons with energies ranging from approximately 1 to 100 keV. In the separator reactor, the radiation interacts with the individual atoms of the flue gas stream. For this energy range, the dominant process is the so-called photoelectric effect, in which the atom absorbs the radiation and an electron is knocked out of the atom. Thus, a positive ion and an electron with energies on the order of a keV unit are produced. This energetic electron can further ionize the surrounding atoms to form “secondary” ions and electrons. As radiation propagates from a source, it is absorbed by the atoms of the substance through which it passes, with the lower-energy photons being the most strongly attenuated. The energy spectrum is weak at distances of centimeters away from the source, particularly in the 1–10 keV region. Specifically, there is a need to ensure that radiation is not shielded in the 1–20 keV energy range, which is the energy of mercury ionization’s maximum efficiency.

Section C in the graph is designated as a cooling period for the X-ray source, during which both the power generator and X-ray source are deactivated. The graph shows a sharp rise in the flow of mercury contents during this period. The ON and OFF methods demonstrate the ability of the entire adsorption system to stimulate the mercury atoms and drive them towards the negative electrode, where the PA6/CS nanofiber is located.

The data about the measurement of mercury contents adsorbed by the PA6/CS nanofibers using the AMA-254 mercury analyzer are presented in Table 3. The quantities of mercury were measured and expressed in nanograms (ng Hg).

The amounts of mercury in the nanofiber samples ranged from 0.2 to 10.9 ng Hg. The presence of Hg on the nanofibers provides evidence that the custom-designed mercury adsorption system and PA6/CS nanofibers effectively collect mercury in gaseous settings. Nevertheless, the measured concentration of Hg on the nanofibers is significantly lower than the quantity of Hg shown in Figure 8. It shows that 10 micrograms/m^3^ of Hg is retained by the electrode every minute, which amounts to 1000 nanograms over 20 min. This can be explained by the fact that most of the directed Hg in section B was mainly adsorbed on the surface of nanofibers by physical means due to X-ray radiation and the electrical field. However, the physically adsorbed Hg is subsequently removed during the cooling stage in section C. The maximum concentration of Hg observed at the beginning of section C, after section B, strongly corroborates this phenomenon. Moreover, X-ray irradiation and electrical fields only affect 5% of the Hg present in the gas. In order to maximize the use of X-rays, it is necessary to locate the X-ray source as close as possible to the reactor with the flue gas. Wide scalability of the radiation intensity is essential to allow for specific plant parameters such as the heavy metal concentration in the flue gas, chamber size, or total flue gas flow through the chamber.

## 4. Conclusions

A custom-made mercury adsorption system was constructed to capture Hg^+^ from gaseous mercury. The X-ray irradiation effectively caused ionization of the elemental mercury, with the electrical field specifically targeting Hg^+^. In an interval of 20 min, 1000 nanograms of mercury was induced to react in the gas and subsequently adhered to the surface of PA6/CS nanofibers by physical adsorption. The mercury inspection of the nanofibers revealed that a total of 11.96 nanograms of mercury was chemically adsorbed, potentially forming bonds with the amino and carboxyl groups of the chitosan nanofiber. The following aspects are suggested to optimize the system and increase the adsorption of mercury:

- Increasing the concentration of chitosan in the nanofibers’ matrix to increase the chemical adsorption of mercury.
- Increasing the size of the nanofiber sample and the size of the electrode in the gas steam.
- Using a stronger X-ray device to induce a higher amount of mercury.
- Modification of the system, which will allow to remove contaminated nanofibers from the cell before the cooling step.
- The potential limitations of the current work are listed as follows:
- The amount of adsorbed mercury is relatively low. Increasing the amount of chitosan molecules in the polymer solution and turning them into a nanofiber form is a sensitive and long optimization procedure. This task may need significant effort and a considerable amount of time.
- Installing a sophisticated and revolutionary filtering device in a coal-fired power station with an X-ray source and electrodes to create an electrical field is difficult due to the presence of solid and old-fashioned structures in the flue gas hazardous reduction system after the combustion process.
- Setting up X-ray irradiation equipment can be expensive, requiring significant capital investment. Handling X-ray equipment requires stringent safety protocols to protect workers from radiation exposure.

In comparison to other existing methods, the offered methodology has the following advantages:

- Electrospun nanofibers provide a large surface area-to-volume ratio, increasing the adsorption sites for mercury.
- Nylon and chitosan are environmentally friendly materials. Chitosan, being a natural biopolymer, is biodegradable. This method minimizes the need for chemical reagents, making the process more environmentally safe.

## Figures and Tables

**Table 1:** Solution and process parameters for electrospinning.

	Value	Unit
Solution Parameters		
PA6/CS concentration in AA/FA (2:1)	10 (9.9:0.1)	(*w*/*w*) %
Process parameters		
Humidity	35	% Rh
Temperature	22	°C
Applied voltage on the lower electrode	−15	kV
Applied voltage on the upper electrode	+65	kV
Distance between electrode	180	mm
Air input	70.6	m^3^/h
Air output	80.4	m^3^/h
Rewinding speed	12	mm/min
Polymer carriage speed	350	mm/s

**Table 2:** EDS analysis of PA6/CS nanofibers.

Element	Electron Transition	Wt. %	Wt. % Deviation	At %
C	K	64.6	0.7	69.9
N	K	15.8	0.8	14.6
O	K	18.5	0.3	15.0
Si	K	0.8	0.0	0.4

**Table 3:** The amount of elemental mercury adsorbed by PA6/CS nanofibers.

Allocated Number of PA6/CS Nanofibers’ Sample	Sample Weight (mg)	Detected Mercury Amount (ng Hg)	Detected Mercury Amount (ng Hg/cm^2^)
1	2.82	0.20	0.03
2	2.55	0.53	0.08
3	2.91	0.33	0.05
4	3.33	10.9	3.28
Total		11.96	

## Data Availability

The original contributions presented in the study are included in the article, further inquiries can be directed to the corresponding author.

## References

[1] Shamshad A., Fulekar M., Bhawana P. (2012). Impact of Coal Based Thermal Power Plant on Environment and Its Mitigation Measure. Int. Res. J. Environ. Sci..

[2] Page A., Elseewi A.A., Straughan I. (1979). Physical and Chemical Properties of Fly Ash from Coal-Fired Power Plants with Reference to Environmental Impacts.

[3] Habib M.A., Khan R. (2021). Environmental Impacts of Coal-Mining and Coal-Fired Power-Plant Activities in a Developing Country with Global Context. Spatial Modeling and Assessment of Environmental Contaminants.

[4] Finkelman R.B. (1999). Trace Elements in Coal: Environmental and Health Significance. Biol. Trace Elem. Res..

[5] Amster E., Lew Levy C. (2019). Impact of Coal-Fired Power Plant Emissions on Children’s Health: A Systematic Review of the Epidemiological Literature. Int. J. Environ. Res. Public Health.

[6] Kravchenko J., Lyerly H.K. (2018). The Impact of Coal-Powered Electrical Plants and Coal Ash Impoundments on the Health of Residential Communities. North Carol. Med. J..

[7] Zhang H., Da Y., Zhang X., Fan J.-L. (2021). The Impacts of Climate Change on Coal-Fired Power Plants: Evidence from China. Energy Environ. Sci..

[8] Widiawaty M.A., Nurhanifah N., Ismail A., Dede M. (2020). The The Impact of Cirebon Coal-Fired Power Plants on Water Quality in Mundu Bay, Cirebon Regency. Sustinere J. Environ. Sustain..

[9] Wang S., Zhang L. (2020). Water Pollution in Coal Wharfs for Coal Loading and Unloading in Coal-Fired Power Plants and Its Countermeasures. J. Coast. Res..

[10] Güleç N., Günal B., Erler A. (2001). Assessment of Soil and Water Contamination around an Ash-Disposal Site: A Case Study from the Seyitömer Coal-Fired Power Plant in Western Turkey. Environ. Geol..

[11] Teng Y., Li P., Wang G., Wang C., Qi N., Zhang K., Wang M. (2023). Effects of SCR Catalyst Breakage on Mercury Emission Properties in Coal-Fired Power Plant: Field Testing and Laboratory Evaluation. Appl. Catal. A Gen..

[12] Li C., Wen C., Wang D., Zhao C., Li R. (2022). Emission Characteristics of Gaseous and Particulate Mercury from a Subcritical Power Plant Co-Firing Coal and Sludge. Atmosphere.

[13] NRDC. https://www.nrdc.org/stories/mercurys-journey-coal-burning-power-plants-your-plate.

[14] Sjostrom S., Durham M., Bustard C.J., Martin C. (2010). Activated Carbon Injection for Mercury Control: Overview. Fuel.

[15] Zhao W., Geng X., Lu J., Duan Y., Liu S., Hu P., Xu Y., Huang Y., Tao J., Gu X. (2021). Mercury Removal Performance of Brominated Biomass Activated Carbon Injection in Simulated and Coal-Fired Flue Gas. Fuel.

[16] Takahashi F., Kida A., Shimaoka T. (2010). Statistical Estimate of Mercury Removal Efficiencies for Air Pollution Control Devices of Municipal Solid Waste Incinerators. Sci. Total Environ..

[17] Pavlish J.H., Sondreal E.A., Mann M.D., Olson E.S., Galbreath K.C., Laudal D.L., Benson S.A. (2003). Status Review of Mercury Control Options for Coal-Fired Power Plants. Fuel Process. Technol..

[18] Kairies C.L., Schroeder K.T., Cardone C.R. (2006). Mercury in Gypsum Produced from Flue Gas Desulfurization. Fuel.

[19] Wo J., Zhang M., Cheng X., Zhong X., Xu J., Xu X. (2009). Hg2+ Reduction and Re-Emission from Simulated Wet Flue Gas Desulfurization Liquors. J. Hazard. Mater..

[20] Zhao S., Peng J., Ge R., Wu S., Zeng K., Huang H., Yang K., Sun Z. (2022). Research Progress on Selective Catalytic Reduction (SCR) Catalysts for NOx Removal from Coal-Fired Flue Gas. Fuel Process. Technol..

[21] Li Y., Yu J., Liu Y., Huang R., Wang Z., Zhao Y. (2022). A Review on Removal of Mercury from Flue Gas Utilizing Existing Air Pollutant Control Devices (APCDs). J. Hazard. Mater..

[22] Kurniawan T.A., Lo W., Liang X., Goh H.H., Othman M.H.D., Chong K.-K., Chew K.W. (2023). Remediation Technologies for Contaminated Groundwater Due to Arsenic (As), Mercury (Hg), and/or Fluoride (F): A Critical Review and Way Forward to Contribute to Carbon Neutrality. Sep. Purif. Technol..

[23] Omer A.M., Dey R., Eltaweil A.S., Abd El-Monaem E.M., Ziora Z.M. (2022). Insights into Recent Advances of Chitosan-Based Adsorbents for Sustainable Removal of Heavy Metals and Anions. Arab. J. Chem..

[24] Haripriyan U., Gopinath K., Arun J. (2022). Chitosan Based Nano Adsorbents and Its Types for Heavy Metal Removal: A Mini Review. Mater. Lett..

[25] Yalcinkaya F. (2019). Preparation of Various Nanofiber Layers Using Wire Electrospinning System. Arab. J. Chem..

[26] Rosli N., Yahya W.Z.N., Wirzal M.D.H. (2022). Crosslinked Chitosan/Poly (Vinyl Alcohol) Nanofibers Functionalized by Ionic Liquid for Heavy Metal Ions Removal. Int. J. Biol. Macromol..

[27] Adil H.I., Thalji M.R., Yasin S.A., Saeed I.A., Assiri M.A., Chong K.F., Ali G.A. (2022). Metal–Organic Frameworks (MOFs) Based Nanofiber Architectures for the Removal of Heavy Metal Ions. RSC Adv..

[28] Haider S., Park S.-Y. (2009). Preparation of the Electrospun Chitosan Nanofibers and Their Applications to the Adsorption of Cu (II) and Pb (II) Ions from an Aqueous Solution. J. Membr. Sci..

[29] Mallik A.K., Kabir S.F., Rahman F.B.A., Sakib M.N., Efty S.S., Rahman M.M. (2022). Cu (II) Removal from Wastewater Using Chitosan-Based Adsorbents: A Review. J. Environ. Chem. Eng..

[30] Algieri C., Chakraborty S., Candamano S. (2021). A Way to Membrane-Based Environmental Remediation for Heavy Metal Removal. Environments.

[31] Escudero-Oñate C., Martínez-Francés E. (2018). A Review of Chitosan-Based Materials for the Removal of Organic Pollution from Water and Bioaugmentation. Chitin-Chitosan - Myriad Functionalities in Science and Technology.

[32] Dhandayuthapani B., Krishnan U.M., Sethuraman S. (2010). Fabrication and Characterization of Chitosan-gelatin Blend Nanofibers for Skin Tissue Engineering. J. Biomed. Mater. Res. Part B Appl. Biomater..

[33] Haaparanta A.-M., Järvinen E., Cengiz I.F., Ellä V., Kokkonen H.T., Kiviranta I., Kellomäki M. (2014). Preparation and Characterization of Collagen/PLA, Chitosan/PLA, and Collagen/Chitosan/PLA Hybrid Scaffolds for Cartilage Tissue Engineering. J. Mater. Sci. Mater. Med..

[34] Prasad T., Shabeena E., Vinod D., Kumary T., Anil Kumar P. (2015). Characterization and in Vitro Evaluation of Electrospun Chitosan/Polycaprolactone Blend Fibrous Mat for Skin Tissue Engineering. J. Mater. Sci. Mater. Med..

[35] Wang Z., Yan F., Pei H., Li J., Cui Z., He B. (2018). Antibacterial and Environmentally Friendly Chitosan/Polyvinyl Alcohol Blend Membranes for Air Filtration. Carbohydr. Polym..

[36] Liu Y., Wang S., Lan W. (2018). Fabrication of Antibacterial Chitosan-PVA Blended Film Using Electrospray Technique for Food Packaging Applications. Int. J. Biol. Macromol..

[37] Kuntzler S.G., Costa J.A.V., de Morais M.G. (2018). Development of Electrospun Nanofibers Containing Chitosan/PEO Blend and Phenolic Compounds with Antibacterial Activity. Int. J. Biol. Macromol..

[38] Zarayneh S., Sepahi A.A., Jonoobi M., Rasouli H. (2018). Comparative Antibacterial Effects of Cellulose Nanofiber, Chitosan Nanofiber, Chitosan/Cellulose Combination and Chitosan Alone against Bacterial Contamination of Iranian Banknotes. Int. J. Biol. Macromol..

[39] Kim S.S., Lee J. (2014). Antibacterial Activity of Polyacrylonitrile–Chitosan Electrospun Nanofibers. Carbohydr. Polym..

[40] Christou C., Philippou K., Krasia-Christoforou T., Pashalidis I. (2019). Uranium Adsorption by Polyvinylpyrrolidone/Chitosan Blended Nanofibers. Carbohydr. Polym..

[41] Ghani M., Gharehaghaji A.A., Arami M., Takhtkuse N., Rezaei B. (2014). Fabrication of Electrospun Polyamide-6/Chitosan Nanofibrous Membrane toward Anionic Dyes Removal. J. Nanotechnol..

[42] Yalcinkaya B., Yalcinkaya F., Chaloupek J. (2016). Thin Film Nanofibrous Composite Membrane for Dead-End Seawater Desalination. J. Nanomater..

[43] Wang S., Hui-Min W., Zhu F.-H., Chen H., Sun X.-L., Zuo Y., Li G. (2011). Mercury Emission Characteristics from Coal-Fired Power Plants Based on Actual Measurement. Huan Jing Ke Xue.

[44] Xie G., Liu Z., Zhu Z., Liu Q., Ge J., Huang Z. (2004). Simultaneous Removal of SO_2_ and NOx from Flue Gas Using a CuO/Al_2_O_3_ Catalyst Sorbent: I. Deactivation of SCR Activity by SO_2_ at Low Temperatures. J. Catal..

[45] Zheng C., Luo C., Liu Y., Wang Y., Lu Y., Qu R., Zhang Y., Gao X. (2020). Experimental Study on the Removal of SO3 from Coal-Fired Flue Gas by Alkaline Sorbent. Fuel.

[46] Al-Deyab S.S., El-Newehy M.H., Nirmala R., Abdel-Megeed A., Kim H.Y. (2013). Preparation of Nylon-6/Chitosan Composites by Nanospider Technology and Their Use as Candidate for Antibacterial Agents. Korean J. Chem. Eng..

[47] Kummer G., Schonhart C., Fernandes M., Dotto G., Missio A., Bertuol D., Tanabe E. (2018). Development of Nanofibers Composed of Chitosan/Nylon 6 and Tannin/Nylon 6 for Effective Adsorption of Cr (VI). J. Polym. Environ..

[48] Nirmala R., Navamathavan R., El-Newehy M.H., Kim H.Y. (2011). Preparation and Characterization of Electrospun Ultrafine Polyamide-6 Nanofibers. Polym. Int..

[49] Qua E., Hornsby P. (2011). Preparation and Characterisation of Nanocellulose Reinforced Polyamide-6. Plast. Rubber Compos..

[50] Yang W., Li R., Fang C., Hao W. (2019). Surface Modification of Polyamide Nanofiber Membranes by Polyurethane to Simultaneously Improve Their Mechanical Strength and Hydrophobicity for Breathable and Waterproof Applications. Prog. Org. Coat..

[51] Shrestha B.K., Mousa H.M., Tiwari A.P., Ko S.W., Park C.H., Kim C.S. (2016). Development of Polyamide-6, 6/Chitosan Electrospun Hybrid Nanofibrous Scaffolds for Tissue Engineering Application. Carbohydr. Polym..

[52] Aridi A., Yusof Y., Chin N., Ishak N., Yusof N., Manaf Y. (2021). Physicochemical Properties of Chitosan Extracted from Leucaena Leucocephala Pods Using Deprotenization and Decolorization Steps.

[53] Gao P., Gao B., Gao J., Zhang K., Yang Y., Chen H. (2016). Chitosan and Its Composites for Removal of Mercury Ion from Aqueous Solution. Prog. Chem..

[54] Kyzas G.Z., Deliyanni E.A. (2013). Mercury (II) Removal with Modified Magnetic Chitosan Adsorbents. Molecules.

